# Targeting the search of African swine fever‐infected wild boar carcasses: A tool for early detection

**DOI:** 10.1111/tbed.14504

**Published:** 2022-03-16

**Authors:** Alberto Allepuz, Mark Hovari, Marius Masiulis, Giovanna Ciaravino, Daniel Beltrán‐Alcrudo

**Affiliations:** ^1^ Department of Animal Health and Anatomy Universitat Autònoma de Barcelona (UAB) Barcelona Spain; ^2^ Food and Agriculture Organization (FAO) Regional Office for Europe and Central Asia Budapest Hungary; ^3^ Emergency Response Division State Food and Veterinary Service Vilnius Lithuania; ^4^ National Food and Veterinary Risk Assessment Institute Vilnius Lithuania; ^5^ Dr. L. Kriauceliunas Small Animal Clinic Veterinary Academy Lithuanian University of Health Sciences Kaunas Lithuania

**Keywords:** African swine fever, early detection, epidemiology, surveillance, wild boar

## Abstract

This study analyses the temporal and spatial distribution of found dead African swine fever (ASF)‐positive wild boar carcasses from 2017 to January 2021 in affected European countries: Bulgaria, Estonia, Germany, Hungary, Latvia, Lithuania, Romania, Poland, Serbia and Slovakia. During this period, a total of 21,785 cases were confirmed in 19,071 unique locations. The temporal analysis of aggregated cases per month evidenced that most countries located in southern latitudes showed a higher number of cases between January and April, whereas in northern latitudes there was no clear temporal pattern. The space–time K‐function evidenced a space–time clustering in the ASF‐positive wild boar carcasses, which was most prominent within distances of 2 km and within 1 week. A Bayesian hierarchical spatial model was calibrated to evaluate the association between the probability of finding ASF‐positive wild boar carcasses and landscape factors (i.e. the presence of a path and paved road), land use and wild boar abundance. Results showed the highest likelihood of finding ASF‐positive wild boar carcasses in areas of transition between woodland and shrub, green urban areas and mixed forests. The presence of a path and a higher abundance of wild boar also increased slightly the odds of finding an ASF‐positive dead wild boar. In summary, this paper aims to provide recommendations to design a search strategy to find ASF‐infected wild boar carcasses, which is a crucial activity in the management of the disease, not just for surveillance purposes (i.e. the early detection of an introduction and the regular monitoring to understand the epidemiology and dynamics), but also for control, namely the disposal of infected carcasses as a virus source.

## 1 INTRODUCTION

African swine fever (ASF) is a disease that affects all members of the *Suidae* family. The disease was first identified in 1921 and since then it has been circulating mainly in Sub‐Saharan Africa. In 1957 and 1960, ASF virus of genotype I arrived to Europe (Spain and Portugal) and then spread to other European countries (Sánchez‐Vizcaino et al., 2013). The disease was eradicated from Europe in 1995 with the exception of the Italian island of Sardinia, which remained endemic (Sauter‐Louis et al., 2021). In 2007, ASF genotype II was introduced into Georgia from where it spread gradually westwards until it reached the European Union (EU) in early 2014, namely Lithuania and Poland (Mačiulskis et al., 2020). Since then, multiple countries in Europe, but also Asia and America have been affected by genotype II with outbreaks in wild boar, domestic pigs or both. The persistence of the disease in wild boar, the lack of an effective vaccine or treatment, and the high case‐fatality rate represent a serious challenge for the global pig sector. At present, biosecurity, movement control and the stamping out of animals are the only tools to fight the disease in domestic pig farms (Sánchez‐Cordón et al., 2018; EFSA et al., 2021).

Finding ASF‐positive wild boar carcasses is a crucial activity in the management of the disease, not just for surveillance purposes, but also for control, namely the disposal of infected carcasses as a source of virus. When it comes to ASF surveillance and early detection in wild boar, it has been repeatedly proven that sampling and testing found dead wild boar is much more efficient than testing hunted wild boar or road kills, even when the later may intuitively seem more convenient. This is explained because the vast majority of wild boar that get infected with the ASF virus will die within days, leaving a very short time window of opportunity to detect the virus in a healthy‐looking animal, i.e. whether incubating animals or the few that survive the infection. Moreover, as soon as wild boars start presenting clinical signs, they tend to hide and rest, which largely prevents them from being hunted. This has very important implications when trying to find the disease in wild boar, both in already infected countries that try to understand the epidemiology and evolution of the disease, but particularly in newly infected countries, where early detection is critical for having a chance at successful control. The active search of carcasses in countries or regions at high risk of ASF, for example, across the border from infected areas, is the most efficient way to early detect the introduction of the disease into ASF‐free wild boar populations.

Wild boar that have died of ASF represent a continuous source of infection for other animals, as the virus might remain infectious in the carcass for an extended period of time, depending on the environmental conditions. It has been reported that a frozen carcass can maintain infectious ASF virus for several months enabling the virus to overwinter and to initiate a new outbreak when the defrosted carcass is visited the following spring by a susceptible wild boar or free‐ranging pig. Therefore, the safe removal of carcasses from the environment and their disposal is an important measure to avoid ASF spread by reducing the local maintenance of the virus (FAO, 2019). The EU developed an ASF strategic approach to prevent and control the spread of the disease and eventually to eradicate ASF from the EU. One of the components of this strategy is finding, testing and disposal of ASF‐infected carcasses (Anonymous, 2020). Optimizing the search (and disposal) of ASF‐infected carcasses should contribute to the eradication of the disease.

However, there are few studies that have attempted to identify in which areas it is more likely to find ASF‐infected carcasses. Similarly, there are currently no instructions or recommendations on where to look for the dead wild boar. The objective of this study was to describe the temporal and space–time distribution of ASF‐positive wild boar carcasses reported from 2017 to January 2021 in Europe and to identify those landscape factors that increase the likelihood of finding these carcasses. This will in turn enable optimization ASF surveillance efforts and strategies.

## 2 MATERIAL AND METHODS

### 2.1 Study area and origin of data

The area of study included the following European countries: Bulgaria, Estonia, Germany, Hungary, Latvia, Lithuania, Romania, Poland, Serbia and Slovakia. Data were provided by the national competent veterinary authorities and covered all ASF‐positive wild boar carcasses found dead, excluding hunted animals and road kills. To ensure that data would be comparable in terms of their quality, spatial resolution and level of detail, only countries reporting to the EU's Animal Diseases Information System (ADIS) were chosen. In fact, all eligible countries under such criteria (i.e. reporting to ADIS and with ASF outbreaks in wild boar) were selected, with the exception of Belgium and the Czech Republic, where, due to the small areas initially affected by ASF, surveillance was particularly intense as compared to other countries. In fact, both countries managed to contain the disease and eventually eradicate it and regain freedom. The Italian island of Sardinia was also excluded because of the different genotype involved.

Data included all eligible events in the target countries. Many countries revised their data collection and only since 2017 they started to provide precise geo‐coordinates for each found dead wild boar. Therefore, the data analysed for countries already affected at the time starts in 2017.

### 2.2 Covariates included in the model

OpenStreetMap data from the area of study was downloaded from Geofabrik (https://www.geofabrik.de/). Paths correspond to those categorized as paths or bridleways in OpenStreetMap. Paved roads included secondary, tertiary and unclassified roads, as well as those with an agricultural use. Water included both water lines (i.e. rivers and streams) and water bodies (i.e. reservoirs or wetlands). Land use was extracted from the Corine Land Cover map, created by the European Environment Agency under the European Union's Earth Observation Programme, named Copernicus [©European Union, Copernicus Land Monitoring Service, 2018, European Environment Agency (EEA)]. This map has a resolution of 100 × 100 m grid and was last updated in 2018. For our study, we used the 44 classes included on level 3 that corresponded to five main land use groups: artificial surfaces, agriculture, forests and semi‐natural areas, wetlands and water bodies. Wild boar abundance was retrieved from the ENETWILD consortium (2020) at a resolution of 2000 × 2000 m grid.

### 2.3 Data management

Data were projected into ETRS89‐extended/LAEA Europe. For each point in which a wild boar had been found dead, the distance to the nearest path, paved road, water line or water body, was calculated by creating a SpatiaLite database for each of these layers. A structured query language (SQL) query was created among them to extract distances. For spatial modelling purposes, a buffer of 2000 m was created around each location in which a wild boar was found dead. The area covered by the buffer was divided into a grid of 500 × 500 m. The locations of ASF‐positive wild boar carcasses were superimposed on this grid and cells that intersected with those points were classified as positive and otherwise, negative. Similarly, paths and paved roads were superimposed on this grid to identify if they were present in each of the grid cells. Water lines and water bodies from OpenStreetMap were not superimposed on this grid, as this land use was already present in the Corine Land Cover map data (i.e. level 3 classes: water courses and water bodies). The zonal statistic plugin was used to obtain the maximum and most frequent values of wild boar abundance and land use, respectively, in that 500 × 500 m grid. All these analyses were done with Quantum GIS 3.18 (QGIS Development Team, 2021).

### 2.4 Temporal analysis and space–time analysis

The forecast library (Hyndman & Khandakar, 2008; Hyndman et al., 2021) in R was used to describe the temporal trend of the number of ASF‐positive wild boar carcasses between January 2017 and January 2021. To construct the time‐series dataset, we used the date when carcasses were confirmed to be infected by ASF by the national reference laboratories. Dates were aggregated by month. The number of cases per month along the different years, and the number of cases found each month in the whole study period were described.

The space–time K‐function, as described by Diggle et al. (2015) was used to describe the excess of risk that could be attributed to an ASF‐positive wild boar carcass as a function of distance and time. In case of no space–time clustering (i.e. when cases occur independently in space and time) the K‐function at each distance and each temporal increase is equal to the product of the K‐function in space and the one in time. The multiplication of the difference between the observed K‐function in space and time by the product of the space and time K‐functions is called the proportional increase in risk or excess of risk due to the presence of space–time interaction. Using the splancs R package, we calculated this value over a space–time grid of 5 km times 2 months using intervals of 500 m and 1 week, respectively. To illustrate any elevated disease risk attributable to space–time interaction this value was plotted as a surface over a space–time grid.

### 2.5 Spatial model

A Bernoulli distribution was used to model the probability of finding ASF‐positive wild boar carcass in each grid cell. The logit transformation was used to link such probability with specific explanatory variables. A backward and forward stepwise procedure based on the Akaike information criterion (AIC) was used to select the best model. Once the best model was selected, it was extended by adding random spatially structured and unstructured components. The spatially structured random effect was defined by a stochastic partial differential (SPDE; Lindgren et al., 2011) and calculated from a matrix of Euclidean distances between grid centroids using Delaunay triangulation (Cameletti et al., 2013; Simpson et al., 2011). This model was solved by using the R‐INLA package (Schrödle & Held, 2011). To assess the association of the variables included in the model with the probability of finding ASF dead wild boar in a grid, 95% credible intervals (CR) were obtained from the exponential of the mean, 2.5% and 97.5% percentiles of the posterior probability distribution of the regression coefficients. We considered a variable to be associated if the probability was over 95%, that is, if the 95% CR was greater or lower than 1. If greater, the variable increased such probability, and if lower, it decreased it.

To validate the ability of the model to discriminate between grids in which it was more likely to find wild boars dead due to ASF, the status (i.e. the classification of a grid as positive or negative) was removed from 30% of randomly selected grid cells. In those cells, the status was predicted by the model and compared with their original value by means of a receiver operating characteristic (ROC) curve constructed using the pROC package (Robin et al., 2011) in R. The area under that curve (AUC) is related to the performance of the model. AUC values greater than .8 and between .7 and .8 are indicative of good and moderate discriminate capacities respectively.

## 3 RESULTS

### 3.1 Descriptive, temporal and space–time analysis results

**FIGURE 1:**
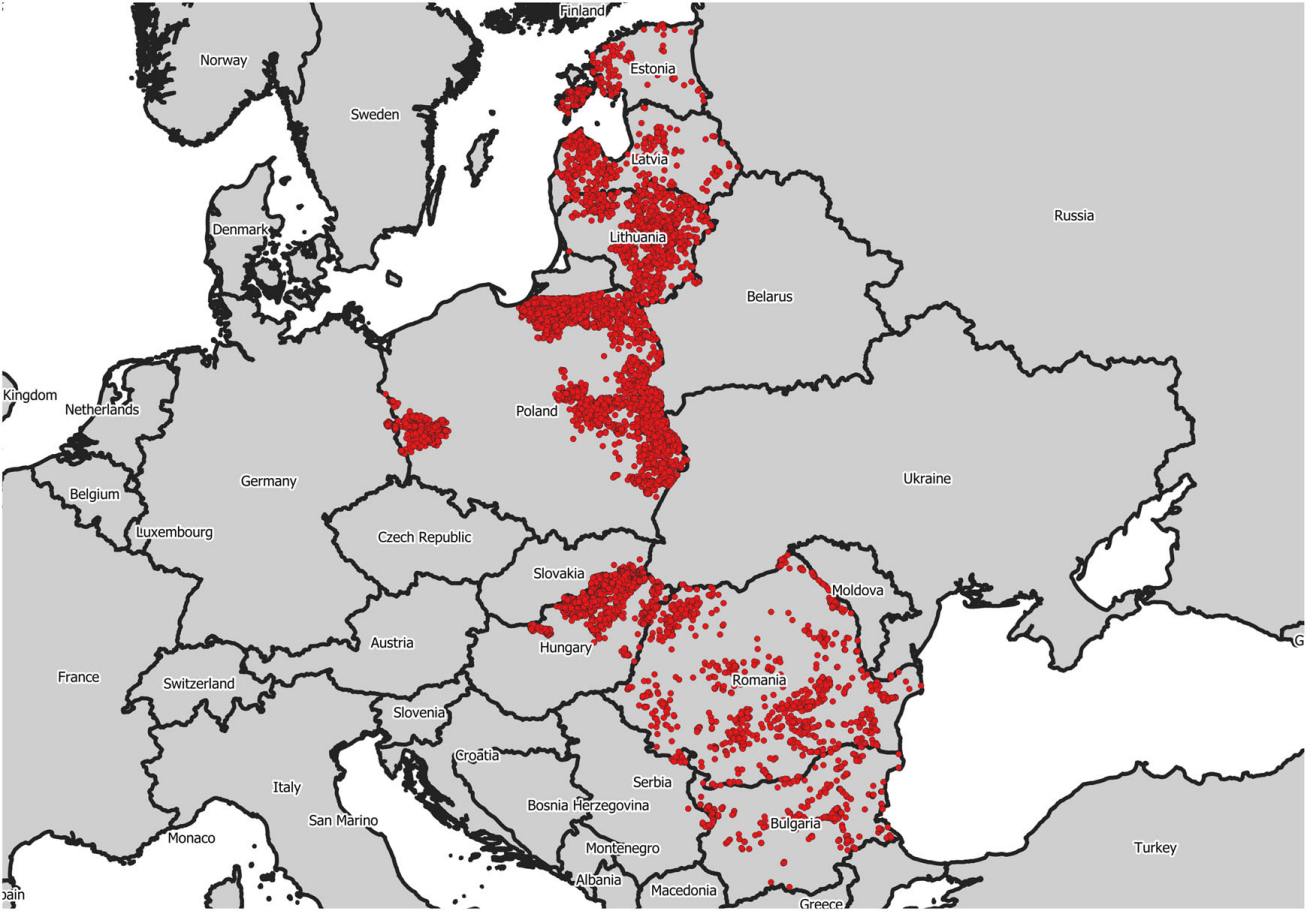
Location of the data used in the study, i.e. African swine fever‐positive wild boar found dead in target countries between January 2017 and January 2021

Table 1 and Figure 1 show the number and location of ASF‐positive wild boars found dead in target countries between January 2017 and January 2021.

**TABLE 1:** Distribution by country and year of the data used in the study, i.e. African swine fever‐positive wild boar found dead in target countries between January 2017 and January 2021

Country	2017	2018	2019	2020	2021	Total
Bulgaria		6	68	980	15	1069
Estonia	185	12	1	6		204
Germany				372	130	502
Hungary		155	2038	4444	57	6694
Latvia	774	269	31	83	6	1163
Lithuania	1065	1035	197	57		2354
Poland	738	2415	2470	2692		8315
Romania		125	385	682	50	1242
Serbia				27	84	111
Slovakia			21	110		131
Total	2762	4017	5211	9453	342	21,785

During this period, a total of 21,758 cases of ASF‐positive in wild boar carcasses were confirmed in 19,071 unique locations (i.e. in some cases, several animals were reported in the same coordinates). The year with more detected cases was 2020. Poland, followed by Hungary, detected the most positives.

**FIGURE 2:**
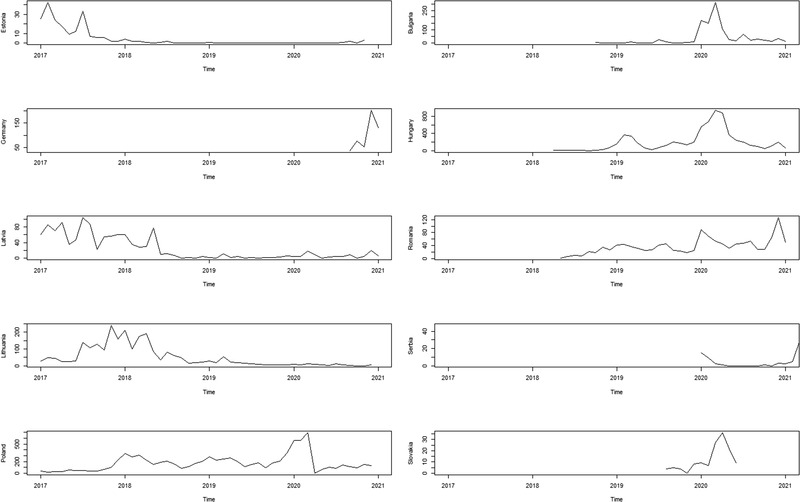
African swine fever‐positive wild boar found dead in target countries per month (January 2017 to January 2021)

The number of ASF‐positive wild boar carcasses per month between 2017 and 2021 in the target countries included in this study can be found in Figure 2.

**FIGURE 3:**
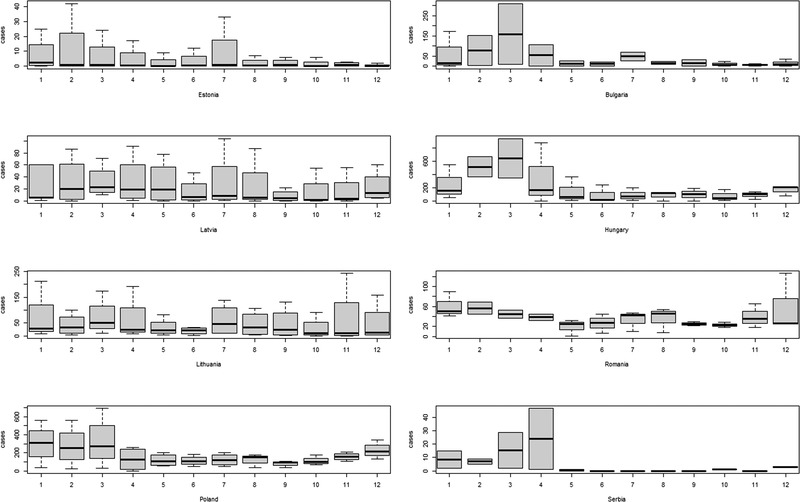
Number of African swine fever‐positive wild boars found dead in target countries per month. Germany and Slovakia are not shown due to the low temporal frame for which cases have been found

The temporal pattern of each country was heterogeneous. Despite this apparent heterogeneity, when plotting cumulative cases per month in the whole period of study (Figure 3), there was a pattern whereby countries in southern latitudes (i.e. Bulgaria, Hungary, Romania and Serbia) showed a higher number of cases from January to April, with the exception of Romania, where there was also a high number of cases in November–December. On the other hand, there was no clear temporal pattern for countries in northern latitudes (i.e. Estonia, Latvia, Lithuania or Poland), with the exception of Poland, where the numbers of cases were slightly higher in winter.

**FIGURE 4:**
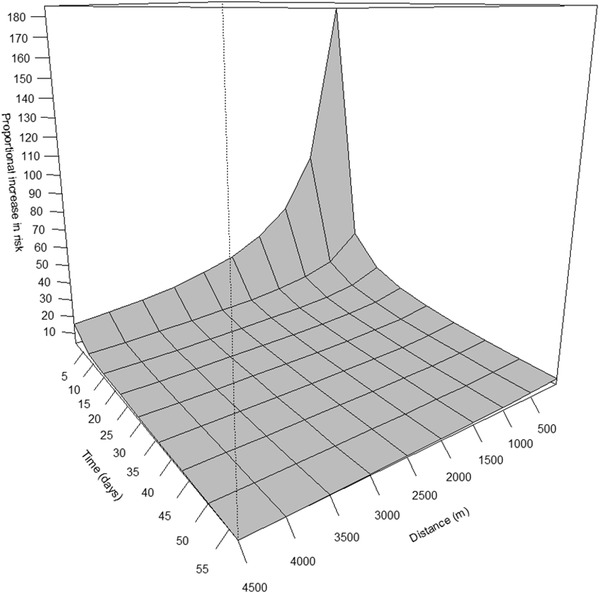
Proportional increase in risk due to space–time clustering with the K‐function. The elevated surface illustrates the excess in risk for finding African swine fever‐positive dead wild boar

Figure 4 shows the plot of the proportional increase in risk from the space–time K‐function. This plot evidences the existence of space–time clustering in the data, which translates in an increase in risk, which is most prominent within 2000 m and within 1 week.

### 3.2 Spatial model results

Table 2 shows the distance between ASF‐positive wild boar carcasses to the nearest path, paved road, water line or water body.

**TABLE 2:** Distance (in meters) from each African swine fever‐positive wild boar carcass to the nearest path, paved road, water line or water body

	Min	Q1	Median	Q3	Max
Path	0	755	2136	4376	22,634
Water*	0	298	887	1759	17,374
Paved	0	161	394	800	8049

Min: minimum; Q1: first quartile; Q3: third quartile; Max: maximum.

* The shortest distance to a water line or water body.

Twenty five per cent of carcasses were found within 755, 161 and 298 m of a path, paved road or water line/body, respectively. Tables 3 and 4 show the most frequent land use in each grid cell together with the presence of a path or paved road and the abundance of wild boar according to the presence or not of ASF‐positive wild boar carcasses in them.

**TABLE 3:** Most frequent land use and presence of a paved road or path in grid cells where African swine fever‐positive wild boar carcasses were found (i.e. positive), versus neighbouring cells in which they were not found (i.e. negative)

Variable	Category	Pos	Neg	Proportion (%)
Land use	Green urban areas	16	128	11.1
	Transitional woodland‐shrub	1107	14,224	7.2
	Mixed forest	2197	30,485	6.7
	Broad‐leaved forest	3677	54,526	6.3
	Sport and leisure facilities	37	566	6.1
	Water courses	52	1012	4.9
	Coniferous forest	1790	35,995	4.7
	Inland marshes	82	1827	4.3
	Land principally occupied by agriculture, with significant areas of natural vegetation	626	14,608	4.1
	Vineyards	66	1567	4
	Discontinuous urban fabric	450	11,063	3.9
	Pastures	1133	30,659	3.6
	Mineral extraction sites	11	312	3.4
	Industrial or commercial units	45	1295	3.4
	Fruit trees and berry plantations	101	2991	3.3
	Complex cultivation patterns	325	11,752	2.7
	Natural grasslands	62	2262	2.7
	Non‐irrigated arable land	2868	114,139	2.5
	Freq_low^a^	15	842	2.3
	Water bodies	79	3817	2
	Rice fields	9	463	1.9
	Peat bogs	12	851	1.4
Paved	Yes	6322	144,437	4.19
	No	8439	190,946	4.23
Path	Yes	1726	21,207	7.53
	No	13,035	314,176	3.98

^a^ Land uses with less than 200 observations in the dataset were grouped in this category.

**TABLE 4:** Descriptive statistics on wild boar abundance in grid cells with and without African swine fever‐positive wild boar carcasses

	*N*	Mean	SD	Min	Q1	Median	Q3	Max
Positive (with)	14,841	54.8	24.7	3.7	36.2	47.2	67.8	181.3
Negative (without)	339,186	49.3	22.9	2.7	33.8	41.9	58.8	181.3

Green urban areas, transitional woodland‐shrub areas, mixed and broad‐leaved forests and sport/leisure areas were the land uses with a higher proportion of positive grid cells. Wild boars were only slightly more abundant in those grids in which dead ASF‐positive wild boar had been found (versus those without).

Odds ratio and their 95% credible intervals (CI) for each of the risk factors from the hierarchical Bayesian model together with the random effects are presented in Table 5.

**TABLE 5:** Fixed and random effects included in the hierarchical Bayesian model, odds ratio (OR), standard deviations (SD) and their 95% credible intervals (CI)

		OR	SD	Credible 2.5%	Credible 97.5%
Land use^a^	Transitional woodland‐shrub	3.1	0.0492	2.8	3.4
	Green urban areas	3.0	0.3670	1.4	6
	Mixed forest	2.9	0.0395	2.7	3.2
	Broad‐leaved forest	2.5	0.0377	2.3	2.7
	Inland marshes	2.4	0.1449	1.8	3.2
	Coniferous forest	2	0.0444	1.8	2.2
	Land principally occupied by agriculture, with significant areas of natural areas	2.0	0.0567	1.8	2.2
	Water courses	1.7	0.1877	1.2	2.5
	Natural grasslands	1.6	0.1604	1.1	2.1
	Pastures	1.5	0.0456	1.4	1.7
	Rice fields	1.5	0.3857	0.7	3.1
	Sport and leisure facilities	1.5	0.2503	0.9	2.4
	Mineral extraction sites	1.5	0.3725	0.7	2.9
	Industrial or commercial units	1.4	0.1865	1	2
	Vineyards	1.4	0.1718	1	1.9
	Complex cultivation patterns	1.2	0.0746	1.1	1.4
	Discontinuous urban fabric	1.2	0.0702	1.1	1.4
	Fruit trees and berry plantation	1.0	0.1441	0.8	1.4
	Water bodies	0.9	0.1466	0.7	1.3
	Freq_low^b^	0.8	0.3072	0.4	1.4
	Peat bogs	0.8	0.3890	0.3	1.5
Path^c^	Path presence	1.1	0.0414	1	1.2
	WBq2^e^	1.1	0.0377	1.1	1.2
Wild boars abundance^d^	WBq3^e^	1.3	0.0390	1.2	1.4
	WBq4^e^	1.3	0.0440	1.2	1.4

		Coefficients	SD		
Random effects	Spatial‐structured random effect	720.9	0.956		
	Non‐spatial structured random effect	0.0106	0.007		

^a^ Reference category was ‘non‐irrigated arable land’.

^b^ Land uses with less than 200 observations in the dataset were grouped in this category.

^c^ Reference category was ‘path absence’.

^d^ Reference category was the first quartile.

^e^ WBq2, WBq3 and WBq4 refer to the second, third and fourth quartile of the wild boar abundance distribution.

Among land use categories, considering non‐irrigated arable lands as the baseline, the model showed the highest likelihood of finding ASF‐positive wild boar carcasses in areas of transition between woodland and shrub, green urban areas and mixed forests, with odds ratios around 3 times higher. The presence of a path and a higher abundance of wild boar also increased slightly the odds of finding an ASF‐positive dead wild boar. On the other hand, the presence of paved roads was not retained in the model as it did not influence the likelihood of finding ASF‐positive carcasses.

**FIGURE 5:**
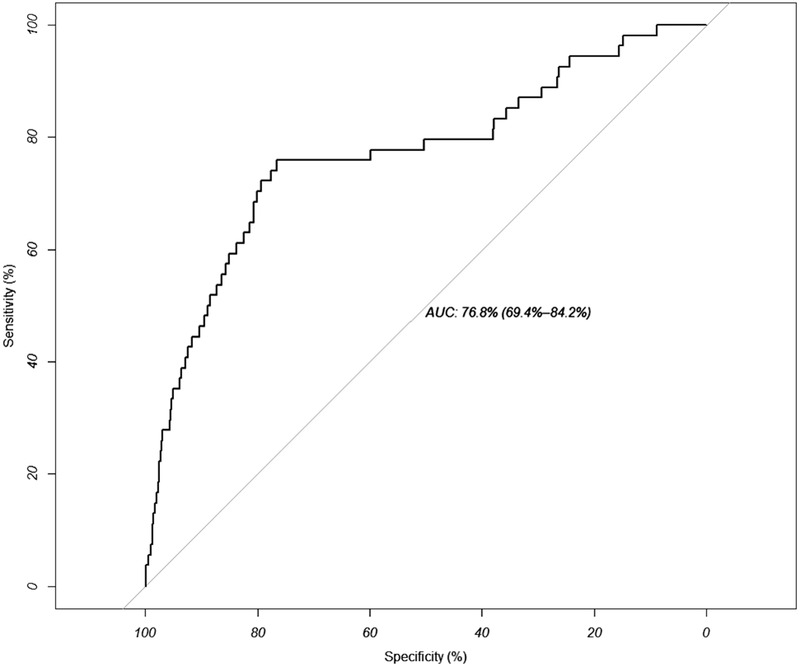
Receiver operating characteristic (ROC) curve to test the ability of the model to discriminate between positive and negative African swine fever grid cells in the 30% of randomly selected cells in which their status against ASF was removed. AUC: area under the curve

For model validation, we randomly selected 30% of cells in which we removed the ASF status and estimated the area under the ROC (receiver operating characteristic) curve (i.e. AUC). Results showed 77% (IC95%: 69.4%–84.2%), indicative of a model with a moderate capacity to discriminate between ASF‐positive and ASF‐negative grid cells (Figure 5).

## 4 DISCUSSION

Early detection is of paramount importance to contain any outbreak. It applies to all transboundary diseases and to both livestock and wildlife. Two key control measures recommended for ASF in wild boar are the active search of dead wild boar and the subsequent disposal of infected carcasses. Results from our study might contribute to increase the efficiency of the search of infected carcasses by allowing to target those areas in which it is more likely to find ASF‐positive dead wild boars. Results from this study showed that some landscape factors (and wild boar abundance to a lesser degree) increased the likelihood of finding ASF‐positive wild boar carcasses and could therefore be used to map those areas that should be prioritized to search for them. In the Czech Republic, Cukor et al. (2020) also attempted to identify those factors linked to the location in which ASF‐positive wild boar carcasses were found. In their study, they determined that most ASF‐infected carcasses were found in forest and especially in young forest areas. These results were explained by the fact that wild boars may choose such areas to die, since they offer silence, cover and lower densities of other animal species. Similarly, our model also showed higher odds of finding ASF‐infected carcasses in certain forests (i.e. mixed, broad‐leaved or coniferous) and areas of transition between woodlands and shrub, which consist of young plants. Moreover, studies conducted in Poland have also identified woodlands as areas with a risk of ASF occurrence (Podgórski et al., 2020). Therefore, these types of land use should be targeted in the search of ASF‐infected carcasses.

Searching near water courses or water bodies has also been recommended, as infected wild boar, when developing clinical signs such as fever and dehydration, search for humid environments and water (Podgórski et al., 2020). Indeed, Cukor et al. (2020) described that around 60% of ASF‐infected carcasses were found up to 100 m from water sources. However, we did not find such a clear association, as only 25% of the ASF‐positive carcasses were found within 298 m from water sources. The association with distance to water might be influenced by other factors, such as temperature. The probability of finding an ASF‐infected carcass near water might be higher during the hotter periods of the year, when animals need more drinking water and cooler resting places, often associated to water sources. Perhaps also the abundance of water (streams and rivers) is important and animals in more arid areas may tend to remain closer to the water. Consequently, the recommendation for searching near water sources might depend on the period of the year and on the land uses present in the target area.

Other landscapes such as green urban areas (OR of 3.0) and sport and leisure facilities (OR of 1.5), or the presence of a path in the grid (OR of 1.1), were also highlighted by the model as areas where it is more likely to find ASF‐infected carcasses. Probably these results respond to the higher human activity, which implies that any wild boar carcass will most likely be quickly found, rather than a predilection of wild boar for those areas. An association between human population density and the number of reports of ASF‐positive carcasses has indeed been reported elsewhere (Lim et al., 2021). Therefore, and despite ASF‐positive wild boar carcasses might be found in these areas, these landscapes should probably not be targeted to search for ASF‐infected carcasses, since they are already indirectly found by people passing by, but rather increase the incentives of the public to report found dead wild boar. Citizen science and mobile application easing such public reporting can assist detection efforts. Accordingly, a participatory workshop with different experts in the field (Jori et al., 2020) highlighted that good communication and transparent information directed to the public was a powerful tool for improving passive surveillance against ASF.

The size of the infected area is another important factor that influences the search of wild boar carcasses once ASFV has been confirmed in an area. The size of the infected area may vary greatly, which makes the targeted search for dead wild boar very demanding in terms of time and human resources. The minimum size of the infected area should be defined based on the geographical distribution of the disease, the wild boar population in the area and the presence of major natural or artificial obstacles to the movements of wild boars. Therefore, it can vary from a few square kilometres to even an entire country. Since the search is time and resource consuming, it is critical to define the area and period of time in which such search should be performed, to optimize the most likely time and location. The space–time analysis evidenced that after the first detection of an ASF‐infected wild boar in an area, the probability of finding ASF‐positive carcasses was higher up to 2 km and over the following week. This combination will offer the best effort‐success ratio. Indeed, many ASF‐affected countries have guidelines for the search of newly infected areas that recommend searching for at least 30 days and focusing on the wild boar feeding and resting places or water sources. The reasoning behind the 1 week temporal pattern might be explained by the fact that wild boar are social animals who live in groups. Most times, several members of the same group will become infected by ASF at about the same time. This implies they will all be dying clustered at approximately the same time (i.e. 1 week) and around the same area (2 km radius). Combining the search in this spatial and temporal frame (focusing on the landscapes identified by the model) with other methods such as the use of hunting dogs (Jori et al., 2020) or drones might also maximize the probability of carcass detection.

As highlighted by the human density factor mentioned above, it is important to stress that this model does not always point to the areas with more ASF‐positive wild boar carcasses, but rather at the places where such carcasses are more easily found, for example, close to paths, in areas often visited by people, and where vegetation is lower and/or thinner, thus allowing for a better visibility. While efforts were done to utilize only data of high quality, by targeting countries that all collect and report data with precise geo coordinates and the same reporting standards/requirements (AIDS), there are a number of biases that are difficult or often impossible to avoid. Perhaps the most important bias relates to the nature of wild boar as a wildlife species, that is, the fact that they live freely, in unknown numbers and densities and without movement restrictions. This implies that finding their carcasses when they die of ASF or any other diseases is a challenging process that translates in a high (but variable) degree of under‐reporting, which will depend on the search effort (whether active or passive), but also on the type of land (e.g. how accessible it is or how thick is the vegetation). These will vary greatly between and even within countries. Efforts were taken when selecting the targeted countries, by avoiding countries with very intensive search effort like the Czech Republic or Belgium. The limited fenced infected area in these two countries allowed a clear shot at eradication (as it indeed happened), which translated in an active search of carcasses that probably lead to the detection of the majority of existing ASF‐positive carcasses in the area. On the other hand, countries with less resources and no economic incentives for the reporting of carcasses were also excluded from the study (i.e. most countries outside the EU, except for Serbia), as the underreporting is considered to be more severe than in study countries. Wild boar management is another important factor, for example, the type of hunting (driven or not), the ban of supplementary feeding, the level of hunting biosecurity, the awareness and cooperation of hunters, the magnitude of (economic) incentives to report, etc. All these differ between and even within countries and affect the way ASF spreads in wild boar population and the chances of finding wild boar. Finally, ecological and climatic factors will also affect the wild boar populations, not just in their abundance (which was accounted through the use of wild boar abundance variable), but also their movement patterns, behaviour and interactions. Factors related to the disease also need to be accounted for. Although all countries are affected by the same genotype (II), there are various strains circulating (Nurmoja et al., 2017), and different levels of endemicity, which translate in different clinical presentations, lethality and other epidemiological parameters. Also, the ASF status in domestic pigs (which may allow the disease to jump back and forward between domestic and wild populations) and other epidemiological factors cannot be excluded as potential biases.

## 5 CONCLUSION

Finding ASF‐positive wild boar carcasses is a crucial activity in the management of the disease, not just for surveillance purposes (i.e. the early detection of an introduction and the regular monitoring to understand the epidemiology and dynamics), but also for control, namely the disposal of infected carcasses as a source of virus. This study, based on thousands of observations, can be translated into very practical applications in the early detection of ASF in wild boar populations. This is key to have a chance at the control and eradication of the disease in wild boar populations, which is otherwise extremely difficult and resource‐consuming. Results pointed that efforts to find (and remove) additional ASF‐positive wild boar carcasses after a confirmed case should be devoted up to 2 km and over the following week. In addition, the model allows to generate search maps or strategies for wild boar carcasses, which focus on the areas with a higher likelihood to find an ASF‐positive wild boar carcass. Rather than covering whole territories, both the generation of maps and the subsequent search efforts should be based on risk assessment approach. Results also helps emergency preparedness to make better simulation exercises for ASF in wild boar, by aiding to better determine where dead wild boar might be found.

For free countries, the mapped areas should be those at a higher risk for ASF introduction, for example, border areas or specific hunting grounds. For infected countries, the rapid finding and subsequent disposal of ASF‐positive wild boar carcasses is one of the key recommended measures to reduce the viral load in the ecosystem, which will eventually translate in less spread of the disease and even its control and eradication.

Easier than generating risk maps is the standardization of search parameters. Already described within the paper, just providing the key risk factors to hunting ground managers is a simple, yet powerful tool to focus search efforts where there are more chances of success, that is, finding an ASF‐positive wild boar carcass. The most important factors identified by the model are (in order of importance):

1. Transitional woodland‐shrub
2. Mixed forest
3. Broad‐leaved forest
4. Inland marshes
5. Coniferous forest
6. Land principally occupied by agriculture
7. Water courses
8. Natural grasslands
9. Pastures
10. Rice fields
11. Vineyards
12. Areas with the highest wild boar density

When trying to find carcasses around an already confirmed ASF‐infected wild boar, active searches should take place within 1 week after the event and in a 2 km radius, focusing in those areas in which is more likely to find them.

## ETHICS STATEMENT

No animals were used in this project.

## Data Availability

The data that support the findings of this study are available on request from the corresponding author. The data are not publicly available due to privacy or ethical restrictions.

## References

[tbed14504-bib-0001] Anonymous . (2020). Strategic approach to the management of African Swine Fever for the EU, Working Document SANTE/7113/2015‐Rev.2 (Accessed: 29.04.2020) https://ec.europa.eu/food/system/files/2020‐04/ad_control‐measures_asf_wrk‐doc‐sante‐2015‐7113.pdf

[tbed14504-bib-0002] Cameletti, M. , Lindgren, F. , Simpson, D. , & Rue, H. (2013). Spatio‐temporal modeling of particulate matter concentration through the SPDE approach. Advances in Statistical Analysis, 97, 109–131. 10.1007/s10182-012-0196-3

[tbed14504-bib-0003] Cukor, J. , Linda, R. , Václavek, P. , Šatrán, P. , Mahlerová, K. , Vacek, Z. , Kunca, T. , & Havránek, F. (2020). Wild boar deathbed choice in relation to ASF: Are there any differences between positive and negative carcasses? Preventive Veterinary Medicine, 177, 104943. 32172021 10.1016/j.prevetmed.2020.104943

[tbed14504-bib-0004] Diggle, P. J. , Chetwynd, A. G. , Häggkvist, R. , & Morris, S. E. (1995). Second‐order analysis of space‐time clustering. Statistical Methods in Medical Research, 4, 124–136. 7582201 10.1177/096228029500400203

[tbed14504-bib-0005] Nielsen, S. S. , Alvarez, J. , Bicout, D. J. , Calistri, P. , Canali, E. , Drewe, J. A. , Garin‐Bastuji, B. , Gonzales Rojas, J. L. , Herskin, M. , Miranda Chueca, M. Á. , Michel, V. , Padalino, B. , Pasquali, P. , Roberts, H. C. , Sihvonen, L. H. , Spoolder, H. , Stahl, K. , Velarde, A. , Viltrop, A. , … Gortázar Schmidt, C. (2021). Scientific Opinion on the African swine fever and outdoor farming of pigs. EFSA Journal, 19, 6639. 10.2903/j.efsa.2021.6639 PMC8188572 34140998

[tbed14504-bib-0006] Acevedo, P. , Croft, S. , Smith, G. C. , Blanco‐Aguiar, O. A. , Fernandez‐Lopez, J. , Scandura, M. , Apollonio, M. , Ferroglio, E. , Keuling, O. , Sange, M. , Zanet, S. , Brivio, F. , Podgórski, T. , Petrović, K. , Soriguer, R. , & Vicente, J. (2020). Validation and inference of high‐resolution information (downscaling) of ENETwild abundance model for wild boar. EFSA Supporting Publication 17, 23. 10.2903/sp.efsa.2020.EN-1787

[tbed14504-bib-0007] FAO, OIE and EC . (2019). African swine fever in wild boar ecology and biosecurity. FAO Animal Production and Health Manual No. 22. Rome.

[tbed14504-bib-0008] Hyndman, R. , Athanasopoulos, G. , Bergmeir, C. , Caceres, G. , Chhay, L. , O'Hara‐Wild, M. , Petropoulos, F. , Razbash, S. , Wang, E. , & Yasmeen, F. (2021). Forecast: Forecasting functions for time series and linear models. R package version 8.15. https://pkg.robjhyndman.com/forecast/

[tbed14504-bib-0009] Hyndman, R. J. , & Khandakar, Y. (2008). Automatic time series forecasting: The forecast package for R. Journal of Statistical Software, 26(3), 1–22. https://www.jstatsoft.org/article/view/v027i03 19777145

[tbed14504-bib-0010] Jori, F. , Chenais, E. , Boinas, F. , Busauskas, P. , Dholllander, S. , Fleischmann, L. , Olsevskis, E. , Rijks, J. M. , Schulz, K. , Thulke, H. H. , Viltrop, A. , & Stahl, K. (2020). Application of the World Café method to discuss the efficiency of African swine fever control strategies in European wild boar (Sus scrofa) populations. Preventive Veterinary Medicine, 185, 105178. 10.1016/j.prevetmed.2020.105178 33099152

[tbed14504-bib-0011] Lim, J. ‐. S. , Vergne, T. , Pak, S.‐I. l , & Kim, E. (2021). Modelling the spatial distribution of ASF‐positive wild boar carcasses in South Korea using 2019–2020 National Surveillance Data. Animals (Basel), 11(11), 1208. 10.3390/ani11051208 33922261 PMC8145688

[tbed14504-bib-0012] Lindgren, F. , Rue, H. , & Lindstrom, J. (2011). An explicit link between Gaussian fields and Gaussian Markov random fields: The SPDE approach (with discussion). Journal of the Royal Statistical Society. Series B, 73, 423–498. 10.1111/j.1467-9868.2011.00777

[tbed14504-bib-0013] Mačiulskis, P. , Masiulis, M. , Pridotkas, G. , Buitkuvienė, J. , Jurgelevičius, V. , Jacevičienė, I. , Zagrabskaitė, R. , Zani, L. , & Pilevičienė, S. (2020). The African swine fever epidemic in wild boar (*Sus scrofa*) in Lithuania (2014–2018). Veterinary Sciences, 7, 15. 10.3390/vetsci7010015 32019088 PMC7157679

[tbed14504-bib-0014] Nurmoja, I. , Petrov, A. , Breidenstein, C. , Zani, L. , Forth, J. H. , Beer, M. , Kristian, M. , Viltrop, A. , & Blome, S. (2017). Biological characterization of African swine fever virus genotype II strains from north‐eastern Estonia in European wild boar. Transboundary and Emerging Diseases, 64(6), 2034–2041. 28116841 10.1111/tbed.12614

[tbed14504-bib-0015] Podgórski, T. , Borowik, T. , Łyjak, M. , & Wo´zniakowski, G. (2020). Spatial epidemiology of African swine fever: Host, landscape and anthropogenic drivers of disease occurrence in wild boar. Preventive Veterinary Medicine, 177, 104691. 10.1016/j.prevetmed.2019.104691 31122672

[tbed14504-bib-0016] QGIS.org (2021). QGIS Geographic Information System. QGIS Association. http://www.qgis.org

[tbed14504-bib-0018a] Robin, X. , Turck, N. , Tiberti, N. , Lisacek, F. , Sanchez, J. C. , & Müller, M. (2011). pROC: An open‐source package for R and S+ to analyze and compare ROC curves. BMC Bioinformatics, 12, 77. 10.1186/1471-2105-12-77 21414208 PMC3068975

[tbed14504-bib-0018] Sánchez‐Cordón, P. J. , Montoya, M. , Reis, A. L. , & Dixon, L. K. (2018). African swine fever: A re‐emerging viral disease threatening the global pig industry. The Veterinary Journal, 233, 41–48. 10.1016/j.tvjl.2017.12.025 29486878 PMC5844645

[tbed14504-bib-0019] Sánchez‐Cordón, P. J. , Montoya, M. , Reis, A. L. , & Dixon, L. K. (2013). African swine fever (ASF): Five years around Europe. Veterinary Microbiology, 233, 41–48. 10.1016/j.tvjl.2017.12.025

[tbed14504-bib-0020] Sauter‐Louis, C. , Conraths, F. J. , Probst, C. , Blohm, U. , Schulz, K. , Sehl, J. , Fischer, M. , Forth, J. H. , Zani, L. , Depner, K. , Mettenleiter, T. C. , Beer, M. , & Blome, S. (2021). African Swine Fever in Wild Boar in Europe—A Review. Viruses, 13, 1717. 10.3390/v13091717 34578300 PMC8472013

[tbed14504-bib-0021] Schrödle, B. , & Held, L. (2011). Spatio‐temporal disease mapping using INLA. Environmetrics, 22, 725–734. 10.1002/env.1065

[tbed14504-bib-0021a] Simpson, D. , Illian, J. , Lindgren, F. , Sørbye, S. H. , & Rue, H. (2011). Going off grid: Computationally efficient inference for log‐Gaussian Cox processes. NTNU Technical report, 10.

